# Correction: Evidence for interplay among yeast replicative DNA polymerases alpha, delta and epsilon from studies of exonuclease and polymerase active site mutations

**DOI:** 10.1186/1741-7007-5-27

**Published:** 2007-06-21

**Authors:** Youri I Pavlov, Satoko Maki, Hisaji Maki, Thomas A Kunkel

**Affiliations:** 1 Laboratory of Molecular Genetics, National Institute of Environmental Health Sciences, National Institute of Health, DHHS, Research Triangle Park, NC 27709, USA; 2 Eppley Institute for Research in Cancer and Department of Biochemistry and Molecular Biology, University of Nebraska Medical Center, Omaha, NE 68198, USA; 3 Laboratory of Microbial Molecular Genetics, Graduate School of Biological Sciences, Nara Institute of Science and Technology, Nara 630-01, Japan; 4 Laboratory of Structural Biology, National Institute of Environmental Health Sciences National Institute of Health, DHHS, Research Triangle Park, NC 27709, USA

## Abstract

This article has been published as a correction for an error in the manuscript of Pavlov et al *BMC Biology *2004, 2:11.

This article has been published as a correction for [1].

The legend for figure 2 in the original article states,

"(C) Plot of time-course of DNA synthesis and dTMP turnover by wild-type Pol e. Open circles connected by solid line represent dTMP retained into DNA; open rectangles connected by a dashed line represent excised dTMP.

(D) Plot of time-course of DNA synthesis and dTMP turnover by Y831A Pol e. Symbols are the same as in (C)."

However, this is incorrect and should be revised to the following,

"(C) Plot of time-course of DNA synthesis by wild-type Pol e (open circles connected by solid line) and Y831A Pol e (open rectangles connected by a dashed line). The data are derived from Fig 2A and are based on amount of dTMP incorporated into DNA.

(D) Plot of time-course of dTMP generation by 3'->5' exonuclease activity by wild-type Pol e and Y831A Pol e (symbols are the same as in (C)). The data are derived from Fig 2B and based on amount of dTMP excised from DNA (turnover)."

We apologise for any inconvenience or confusion that this may have caused.

Here we have provided the original figure and the corrected legend, as figure 1.

**Figure 1:**
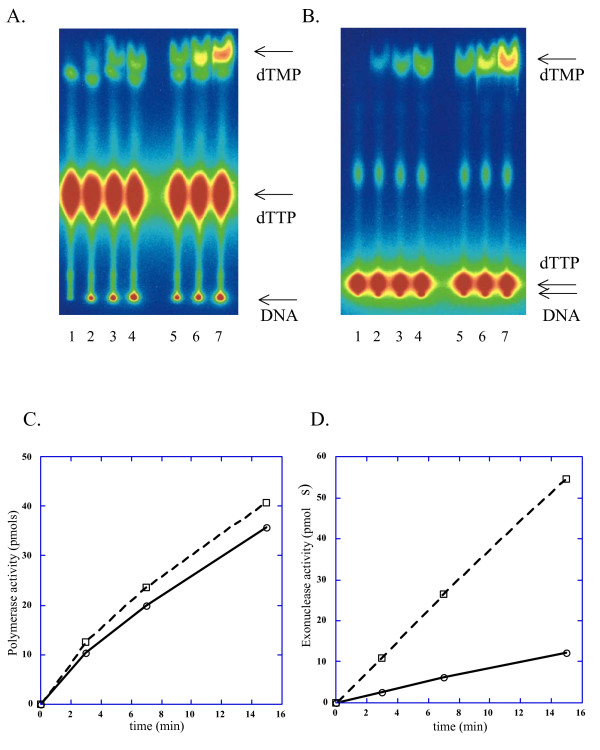
**This is the original image for figure 2, with the corrected version of the legend**. Elevated nucleotide turnover by Y831A Pol ε. DNA synthesis and dTTP turnover by Pol ε was measured on poly(dA)/oligo(dT) substrate. Reactions were performed as described in Methods, using 0.13 U of each enzyme for 20 μl reactions. **(A) **Analysis of polymerase reaction by TLC in 1 M LiCl running buffer. Lanes 1, 2, 3, 4: wild-type Pol ε at 0, 3, 7 and 15 minutes of reaction, respectively. Lanes 5, 6, 7: reactions with Y831A Pol ε at 3, 7 and 15 minutes, respectively. Positions of unincorporated label, label in DNA and dTMP are shown by arrows. **(B) **Analysis of polymerase reaction by TLC in 0.4 M LiCl running buffer. Lane assignment is the same as in (A). **(C) **Plot of time-course of DNA synthesis by wild-type Pol e (open circles connected by solid line) and Y831A Pol e (open rectangles connected by a dashed line). The data are derived from Fig 2A and are based on amount of dTMP incorporated into DNA. **(D) **Plot of time-course of dTMP generation by 3'->5' exonuclease activity by wild-type Pol e and Y831A Pol e (symbols are the same as in (C)). The data are derived from Fig 2B and based on amount of dTMP excised from DNA (turnover).
